# Volumetric Differences in Cerebellar Lobes in Individuals from Multiplex Alcohol Dependence Families and Controls: Their Relationship to Externalizing and Internalizing Disorders and Working Memory

**DOI:** 10.1007/s12311-015-0747-8

**Published:** 2015-11-20

**Authors:** Shirley Y. Hill, Sarah D. Lichenstein, Shuhui Wang, Jessica O’Brien

**Affiliations:** 1 Department of Psychiatry, University of Pittsburgh School of Medicine, 3811 O’ Hara St, Pittsburgh, PA 15213 USA; 2 Department of Psychology, University of Pittsburgh, Pittsburgh, PA USA

## Abstract

Offspring from families with multiple cases of alcohol dependence have a greater likelihood of developing alcohol dependence and related substance use disorders. Greater susceptibility for these disorders may be related to cerebellar morphology. Because posterior regions of the cerebellum are associated with cognitive abilities, we investigated whether high-risk offspring would display regionally specific differences in cerebellar morphology and whether these would be related to working memory performance. The relationship to externalizing and internalizing psychopathology was of interest because cerebellar morphology has previously been associated with a cognitive affective syndrome. A total of 131 participants underwent magnetic resonance imaging (MRI) with volumes of the cerebellar lobes obtained with manual tracing. These individuals were from high-risk (HR) for alcohol dependence families (*N* = 72) or from low-risk (LR) control families (*N* = 59). All were enrolled in a longitudinal follow-up that included repeated clinical assessments during childhood and young-adulthood prior to the scan that provided information on Axis I psychopathology. The Working Memory Index of the Wechsler Memory Scale was given at the time of the scan. Larger volumes of the corpus medullare and inferior posterior lobes and poorer working memory performance were found for the HR offspring relative to LR controls. Across all subjects, a significant positive association between working memory and total volume of corpus of the cerebellum was seen, controlling for familial risk. Presence of an internalizing or externalizing disorder interacting with familial risk was also associated with volume of the corpus medullare.

## Introduction

The cerebellum has long been known to play a critical role in motor control, motor learning, and coordination [1]. More recently, studies have demonstrated that in addition to its known efferents to prefrontal motor regions, the cerebellum also projects to areas of the frontal cortex that have been implicated in cognitive functioning [2]. Additionally, cerebellar involvement in a variety of higher order cognitive functions, including language, visuospatial, executive, and working memory processes, has been demonstrated using functional magnetic resonance imaging (fMRI) [3]. Further, distinct regions of the cerebellum correspond to different functions, with the posterior lobe preferentially involved in cognition [4–6].

In particular, converging evidence from several lines of research supports the importance of the posterior cerebellar lobes for working memory performance. Lesion studies have demonstrated that individuals with posterior cerebellar lesions consistently display working memory deficits [4, 7, 8], whereas damage to other cerebellar regions does not appear to affect working memory [7]. Positive correlations between posterior cerebellar lobe volume and working memory performance have been reported [9–11] along with greater BOLD activation during working memory task performance [12–14].

Cerebellar abnormalities have frequently been reported in alcohol-dependent individuals, presumably in association with the neurotoxic effects of long-term alcohol consumption [6]. Chanraud and colleagues [15] found bilateral reductions in cerebellar gray matter among alcohol-dependent individuals who had been abstinent for approximately 6.5 months. Smaller cerebellum volume predicted poorer performance on executive functioning tasks. Importantly, cerebellar volume did not correlate with the amount of lifetime alcohol consumption, suggesting that reduced cerebellar volume may represent a premorbid risk factor for the development of alcohol abuse, and not merely a consequence of exposure. In fact, age of onset was the only drinking variable that significantly predicted cerebellar morphology in their sample, with earlier age of drinking onset associated with lesser bilateral cerebellum volume. Because early age of onset to drinking is a known characteristic of individuals at high genetic risk for alcoholism [16], these data suggest that cerebellar abnormalities may contribute to a predisposition to alcohol use problems. On the other hand, because those with earlier age of onset will have longer drinking histories at the time they are evaluated than those with later onset, it may be the case that the smaller volume observed in the early onset cases was simply a reflection of greater years of alcohol exposure.

In addition to the structural abnormalities of the cerebellum that have been identified in long-term alcohol-dependent individuals [17], adolescent binge drinkers show reduction in cerebellar volume [18]. Furthermore, aberrant cerebellar volume has been found to correlate with motor deficits among alcoholics, including abnormal postural sway [17, 19] as well as impaired cognitive performance, including poorer executive function, visuospatial ability [17], and spatial working memory performance [9].

A number of studies have identified cerebellar abnormalities among individuals at high-risk for alcoholism by virtue of family history, including aberrant structure [20–22], function [23, 24], and functional connectivity [25]. Furthermore, aberrant brain morphology has been shown to be associated with factors known to increase risk for subsequent alcohol use disorders, including greater impulsivity [26] and increased likelihood of having adolescent externalizing behaviors [22]. Therefore, volumetric aberrations may provide the structural underpinnings of a variety of characteristics that are typical of individuals with a family history of alcohol dependence.

One prominent theory of susceptibility to alcohol dependence suggests that high-risk youngsters may show developmental delays in achieving age appropriate neurobiological milestones including P300 amplitude [27], postural control [28], and pruning of cerebellar gray matter [20, 21]. Accordingly, larger total cerebellar volume [20, 21] among high-risk offspring from families with a high density of alcohol dependence may be indicative of a delay in cerebellar development among this population. Volume of the cerebellum has previously been shown to follow a quadratic developmental trajectory, peaking at 11.8 years in females and 15.6 years in males [29]. Moreover, age-related regression of cerebellar volume in high- and low-risk participants reveals that the quadratic developmental trajectory seen in low-risk control subjects is disrupted by being a member of a high-risk family [23]. Therefore, the larger cerebellar volume observed among high-risk individuals may reflect a delay in brain maturation that may contribute to behavioral characteristics that increase addiction susceptibility. However, one small study of 20 high- and 20 low-risk offspring reported smaller volumes of the right cerebellar hemisphere among high-risk males [22]. Given the proposed topographical organization of the cerebellum, with distinct regions corresponding to different functional domains, it is of interest to investigate whether individuals at high-risk for alcoholism display distinct abnormalities in specific cerebellar lobes.

There is now a substantial literature demonstrating that there are widespread neuropsychological deficits among individuals at high familial risk for alcohol dependence [30, 31], which are thought to reflect heritable biological abnormalities [31]. In particular, working memory deficits have been frequently reported among individuals considered to be at high-risk for developing alcohol dependence (AD) due to their family history of AD [32, 33]. Additionally, aberrant cerebellar activation [24] and functional connectivity [25] during working memory task performance has been seen in high-risk individuals.

The current study was designed to determine whether high-risk offspring would display abnormalities in regions of the cerebellum that have been specifically linked to cognitive functioning, and whether morphology of the posterior cerebellar lobes would predict working memory performance. Also, of interest was the determination of whether the presence of internalizing and externalizing disorder would be associated with volumetric differences especially in view of the greater likelihood that high-risk offspring are significantly more likely to manifest an externalizing disorder that is accompanied by neurobiological indicators of disinhibition [16].

## Methods

### Sample

A total of 131 participants were included in the current analyses, including 72 high-risk (HR) and 59 low-risk (LR) individuals. This sample represents a subset of participants in an ongoing longitudinal study in which offspring from families with a high density of alcohol-dependent members are compared with offspring from control families. HR families were selected based on an adult proband pair of alcohol-dependent brothers. Targeted families were excluded if either member of the proband pair, or any first-degree relative, had a primary diagnosis of recurrent major depressive disorder, bipolar disorder, primary drug dependence, or schizophrenia by DSM-III criteria based on the Diagnostic Interview Schedule (DIS) [34]. Offspring of the proband pair and their siblings were eligible for the longitudinal follow-up. Although some high-risk offspring did not have an alcohol-dependent parent, all of the offspring had a significantly higher density of familial alcohol dependence through aunts, uncles, and grandparents (an average of four first- and second-degree relatives). Control families were selected for a proband pair of same-sex adult siblings. Probands and their first-degree relatives were free of any Axis I psychopathology according to the DIS.

All offspring were eligible for participation in the MRI portion of the study. Subjects were sent letters describing the adjunctive study procedures, and respondents were screened for the presence of ferromagnetic metal in or on their body. Female subjects were also screened for pregnancy using Icon 25 hCG (Beckman Coulter, Fullerton, California) pregnancy kits. The study has ongoing approval from the University of Pittsburgh Institutional Review Board. All participants provided consent at each visit. Children provided assent with parental consent.

### Offspring Assessments

#### Clinical Assessments

Each child/adolescent offspring received an annual clinical assessment for DSM-III diagnoses using the Schedule for Affective Disorders and Schizophrenia (K-SADS) until age 19 [35]. Thereafter, annual clinical follow-ups included the Composite International Diagnostic Interview (CIDI) [36] to determine the presence or absence of DSM-IV diagnoses and the CIDI-Substance Abuse Module (CIDI-SAM) [37] to measure the quantity, frequency, and pattern of substance use. Interrater reliability for diagnostic instruments exceeded 90 %. Repeated assessments throughout childhood and young adulthood (mean = 5.32 ± 4. 11 SE approximately yearly visits) provided the opportunity to determine the age of onset for developing a substance use disorder. This provided covariate information for the statistical analysis aimed at determining the effect of familial risk on regional cerebellar volumes.

Mothers of both the high- and low-risk offspring were administered a structured interview designed to assess the quantity and frequency of use of alcohol, drugs, and cigarettes during pregnancy. The instrument was given at the time of the first longitudinal assessment when the mother accompanied the child to the laboratory. Data were available for 111 of the children for alcohol use, 108 for drug use, and 105 for cigarette use during pregnancy.

#### Neuropsychological Testing

Participants were administered the Wechsler Memory Scale—Third Edition (WMS-III) [38], with age-adjusted scaled scores utilized in the current analyses. Working memory was quantified using the working memory index, calculated from scaled scores for the Letter-Number Sequencing and Spatial Span subtests.

#### Magnetic Resonance Imaging

**Fig. 1:**
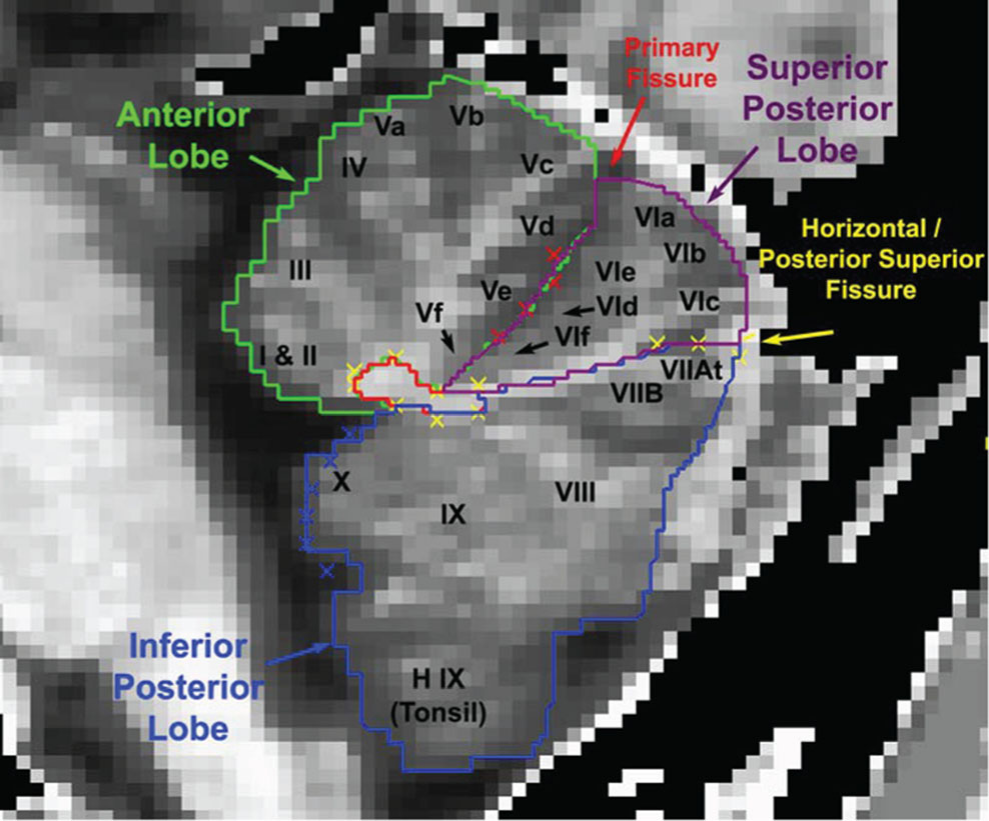
Regions of interest traces are shown in the sagittal plane. Measured ROIs included the anterior, superior posterior, and inferior posterior lobes and the corpus medullare. Figure from Pierson et al. [40]. The *yellow Xs* (*oval cluster*) represent the outline of the corpus medullare with the *remaining yellow Xs* indicating the horizontal fissure

Subjects were scanned on a GE 1.5 Tesla scanner in the Department of Radiology MR Research Center. T1-weighted, T2-weighted, and axial proton density images were obtained, as previously described [21]. Regions of interest were drawn using BRAINS2 [39], a program that uses a semiautomated segmentation approach to provide reliable and valid structural volumetric measurements. Two raters who were blind to subject identity and risk group status traced the volumes of the cerebellar lobes and intracranial volume (ICV) according to the guidelines established by Pierson et al. [40]. The anterior, inferior posterior, and superior posterior lobes were traced along with the corpus medullare. The corpus medullare consists of the central white matter and the output nuclei of the cerebellum. Deep within the corpus medullare, these small gray matter nuclei provide much of the output of the cerebellum (Fig. 1).

### Statistical Analyses

#### Risk Effects on Cerebellar Lobe Volumes

Linear mixed model regression analyses were used to assess the impact of risk group status on cerebellar lobe volumes and working memory performance. Risk, gender, age, and prior substance use disorder diagnosis as well as the interaction between risk and gender were entered as factors and a family identifier was included as a random effect variable to account for the inclusion of multiple siblings.

#### Association Between Cerebellar Lobes and Working Memory

Partial correlations were conducted to assess the associations between working memory task performance and cerebellar lobe volumes. Additionally, risk status and age were included as covariates to clarify the association between volumes of the cerebellar lobes and neuropsychological performance.

#### Influence of Internalizing and Externalizing Psychopathology

Linear mixed model analyses were conducted to determine whether the presence of internalizing or externalizing disorders influences cerebellar lobe volumes and/or working memory performance. Subjects were classified as having an externalizing disorder if they met criteria for attention-deficit hyperactivity disorder (ADHD), conduct disorder, oppositional defiant disorder, or any substance use disorder at any point during the follow-up period. Participants were classified as having an internalizing disorder if they met criteria for major depressive disorder or any anxiety disorder throughout their follow-up. Additionally, in order to partial out the effects of internalizing and externalizing disorders, individuals meeting criteria for disorders in both categories were excluded from the analyses, leaving a total of 53 participants with a single syndrome available for analysis. The presence of lifetime internalizing or externalizing pathology was entered as a predictor variable, with risk, gender, and age as additional factors with a family identifier included as a random effect variable to account for the inclusion of multiple siblings.

## Results

### Sample Characteristics

A total of 131 subjects were included in the current analyses (72 high-risk, 59 low-risk; 66 male, 65 female). The high- and low-risk groups did not differ significantly in gender distribution or scan age. Based on mean levels of socioeconomic status (SES), subjects in the HR group had a significantly lower SES at the time of study entry. However, the mean SES of both risk groups corresponds to the same Hollingshead social stratum, representing medium business, minor professional, and technical workers, suggesting quite similar socioeconomic background.

Consistent with the pattern observed in the larger longitudinal studies [41, 42], the 72 HR individuals had significantly higher rates of psychopathology by the time of their last clinical assessment than did the 59 low-risk controls. Also, a total of 22 participants (18 HR, 4 LR) met criteria for alcohol or drug abuse or dependence prior to the time of their MRI scan. Because exposure to drugs and alcohol is known to have widespread neurotoxic effects [43], analyses examining effects on brain structure included prior SUD diagnosis as a covariate, in order to distinguish between the effects of premorbid genetic risk and substance exposure. See Table 1 for sample characteristics.

**Table 1:** Demographic characteristics of high-risk and low-risk adolescents and young adults

	High-risk (*N* = 72) 38 males and 34 females		Low-risk (*N* = 59) 28 males and 31 females				
	Mean	SD	Mean	SD	*T*	*df*	*p*
Age^a^	18.66	4.25	18.23	5.87	0.47	1, 129	NS
Males—intracranial volume (cc)	1497.18	93.49	1446.21	121.88	1.92	1, 64	NS
Females—intracranial volume (cc)	1339.81	103.6	1279.83	83.63	2.55	1, 63	0.013
BMI male	24.33	5.17	22.90	3.87	1.22	1, 64	NS
BMI female	23.18	4.97	25.63	6.39	1.71	1, 63	NS
IQ male^b^	109.88	15.03	115.97	15.96	1.53	1, 33	NS
IQ female^b^	104.71	15.53	108.34	16.61	0.91	1, 52	NS
SES^c^	40.83	11.26	45.81	9.98	4.98	1, 129	0.009
Number right handed (%)^d^	69 (95.8)		55 (93.2 )		0.44	1, 131	NS
Alcohol or drug abuse/dependence lifetime^e^	37		13		11.84	1, 131	0.001
Alcohol or drug abuse/dependence diagnosis prior to scan^f^	18		4		7.70	1, 131	0.004

^a^Age range for high-risk was 9–28 years and 8–29 years for low-risk controls

^b^IQ was based on Peabody Picture Vocabulary Test. A mixed model analysis of variance controlling for sibling resemblance (random effect) and presence of SUD before time of testing was used

^c^Hollingshead Four Factor Index was used to determine SES. Hollingshead scores are grouped into five groups with group I (scores 8–19) indicating the lowest level of SES, unskilled laborers, and menial service workers through the top category, category V (scores 55–66) indicating Major business owner or professional. Although the group means differed, the SES mean values are within the same Hollingshead group, level IV, medium business owner, minor professional and technical

^d^Hand preference reported by participant

^e^Number of cases meeting criteria for alcohol or drug abuse or dependence during the longitudinal follow up. Diagnoses were made using the age-appropriate diagnostic instrument, KSADS for those under age 19 and CIDI for those 19 or greater

^f^Number of cases meeting criteria for alcohol or drug abuse or dependence prior to the MRI scan

#### Prenatal Use of Substances

Analyses were performed to assess any potential influence of the mothers’ prenatal use of substances. Mothers of both the high- and low-risk offspring reported low levels of drinking, drug, and cigarette use during pregnancy. A total of 98.2 % reported drinking less than one drinking per day during the pregnancy (69.4 % reported no drinking). Absence of cigarette smoking was reported by 76.2 % of the sample. Drug use was reported in 3.7 % of cases.

### Risk Effects on Cerebellar Lobe Volumes

#### Total Cerebellar Lobes

Total cerebellar volume was determined by using the volume of each manually traced cerebellar lobe which was then summed to derive a measure of total cerebellar volume. Total cerebellar lobe volume was larger in high-risk than low-risk offspring (*p* = 0.019).

#### Cerebellar Lobe Volumes

The effects of familial risk group membership, gender, age, and the interaction of risk and gender were assessed for total volume, gray, and white matter volumes of the corpus medullare, superior posterior, inferior posterior, and anterior cerebellar lobes (Table 2). These analyses revealed significant differences by familial risk for total volume, total gray and total white matter for the corpus medullare, and the inferior posterior lobes. Gender effects were observed across all four regions tested for total volumes and for gray matter with females showing lesser volume than males. Age effects were seen for the corpus medullare for both total gray and total white matter (Table 2). Hemispheric differences were assessed for all of the lobes. Significant effects of risk status were observed for the inferior posterior (left: *p* = 0.031, right: *p* = 0.053) lobes and for the total bilateral volume (*p* = 0.030) with high-risk offspring showing greater total volume (63.40 ± 0.87 SE) than the low-risk controls (60.42 ± 0.99 SE). For the corpus medullare, differences were seen in both hemispheres (left: *p* = 0.014, right: *p* = 0.029), with high-risk participants displaying larger corpus medullare volumes in each hemisphere. Bilateral total volume of the corpus medullare was also greater for high-risk offspring (15.53 ± 0.22 SE versus 14.68 ± 0.26 SE).

**Table 2:** Effect of familial risk for alcohol dependence on volume of cerebellar lobes

	Corpus			Anterior			Superior posterior			Inferior posterior		
	*F*	*df*	*p*	*F*	*df*	*p*	*F*	*df*	*p*	*F*	*df*	*p*
Total volume												
Risk	6.03	1, 71.42	0.016	2.02	1, 67.96	–	2.13	1, 62.47	–	4.94	1, 57.57	0.03
Gender	21.82	1, 117.83	0.000	13.77	1, 124.99	0.000	13.40	1, 111.19	0.000	34.38	1, 123.52	0.000
Age	2.17	1, 115.81	–	0.01	1, 104.71	–	0.53	1, 116.09	–	0.44	1, 102.42	–
Risk × gender	0.006	1, 116.86	–	1.75	1, 124.99	–	0.03	1, 109.64	–	0.16	1, 123.12	–
SUD before scan	0.06	1, 124.9	–	0.63	1, 121.78	–	0.02	1, 124.20	–	0.42	1, 123.39	–
Total gray												
Risk	6.49	1, 72.95	0.013	1.40	1, 68.77	–	2.76	1, 62.18	–	3.03	1, 70.80	–
Gender	16.93	1, 123.00	0.000	12.28	1, 122.95	0.001	13.57	1, 104.16	0.000	43.86	1, 120.49	0.000
Age	9.92	1, 109.08	0.002	0.49	1, 107.72	–	0.55	1, 118.47	–	1.74	1, 112.26	–
Risk × gender	0.08	1, 123.00	–	1.74	1, 122.97	–	0.004	1, 102.96	–	0.87	1, 120.35	–
SUD before scan	1.55	1, 119.73	–	0.86	1, 119.71	–	0.17	1, 121.32	–	0.12	1, 122.30	–
Total white												
Risk	4.26	1, 68.85	0.043	0.18	1, 65.31	–	0.96	1, 71.28	–	6.05	1, 123	0.015
Gender	16.34	1, 119.70	0.000	2.76	1, 120.64	–	2.88	1, 122.21	–	0.44	1, 123	–
Age	0.45	1, 112.16	–	2.04	1, 109.73	–	11.02	1, 106.81	0.001	1.60	1, 123	–
Risk × gender	0.02	1, 119.51	–	0.11	1, 120.52	–	0.09	1, 121.98	–	0.13	1, 123	–
SUD before scan	0.01	1, 122.47	–	0.30	1, 121.89	–	0.16	1, 117.48	–	1.56	1, 123	–

#### Risk Effects on Working Memory Performance

Working memory was determined from scaled scores for the Letter-Number Sequencing and Spatial Span subtests. The effect of familial risk was tested to determine its effect on working memory performance with gender included in the model as a covariate. High-risk individuals showed poorer working memory function than low-risk controls (*F* = 5.57, *df* = 1, 56.6, *p* = 0.022).

### Association Between Cerebellar Lobe Volumes and Working Memory

In order to determine if volume of particular cerebellar lobes was associated with working memory performance, partial correlations were conducted to quantify associations between bilateral cerebellar lobe volumes and working memory performance, controlling for risk and gender. These analyses revealed significant positive correlations between Working Memory scaled scores and total bilateral volumes of the corpus medullare and superior posterior lobes, with marginal effects found for the inferior posterior lobe (Table 3).

**Table 3:** Partial correlations between cerebellar lobes and working memory controlling for age and familial risk

	Working memory	Corpus	Inferior/posterior	Anterior	Superior/posterior
Working memory		*r* = 0.203	*r* = 0.176	*r* = 0.122	*r* = 0.235
	–	*p* = 0.031	*p* = 0.062	*p* = NS	*p* = 0.012
Corpus		–	*r* = 0.649	*r* = 0.315	*r* = 0.369
			*p* = 0.000	*p* = 0.001	*p* = 0.000
Inferior/posterior			–	*r* = 0.460	*r* = 0.416
				*p* = 0.000	*p* = 0.000
Anterior				–	*r* = 0.144
					*p* = NS

Positive partial correlations were found for the corpus medullare (*r* = 0.20, *p* = 0.03), superior posterior lobes (*r* = 0.24, *p* = 0.012), and inferior posterior lobes (*r* = 0.18, *p* = 0.06). This relationship was examined further by performing partial correlations between volumes and working memory performance adjusting for age within each risk group. Positive correlations between working memory performance and superior posterior volume were found for the low-risk controls (*r* = 0.38, *df* = 48, *p* = 0.007), but no relationship was seen for the high-risk offspring (*r* = 0.16, *df* = 61, ns). Similarly, working memory performance and volume of the corpus medullare showed a significant positive correlation for the low-risk controls (*r* = 0.32, *df* = 48, *p* = 0.025) but a significant relationship was not seen in the high-risk offspring (*r* = 0.15, *df* = 61, ns).

### Influence of Internalizing and Externalizing Psychopathology

Due to the elevated rate of psychopathology seen among high-risk offspring overall [41, 42], it was of interest to assess whether history of internalizing or externalizing disorders in the present sample that received an MRI scan would be associated with volumes of specific cerebellar lobes or working memory performance. A mixed model analysis was performed to determine if the presence of any externalizing disorder in childhood or young adulthood would be associated with volume of the corpus medullare, anterior, superior posterior, and inferior posterior lobes. The model included covariates for risk, gender, scan age, and presence of any internalizing or externalizing disorder during childhood or young adulthood. No significant main effects of externalizing psychopathology on cerebellar volume were observed, though risk and gender were significant (Tables 4 and 5). However, a significant interaction between risk status and externalizing disorder was observed (*F* = 4.82, *df* = 1, 124.5, *p* = 0.030), with high-risk participants having a history of externalizing disorders showing lesser corpus medullare volume (14.93 ± 0.41) versus those without a history of externalizing psychopathology (15.71 ± 0.24). In contrast, low-risk participants with a history of externalizing diagnoses displayed larger corpus medullare volume relative to those without the presence of an externalizing disorder in either childhood or young adulthood (15.26 ± 0.47 versus 14.51 ± 0.29), respectively. Tests for a potential interaction between familial risk status and having a history of having any internalizing disorder in childhood or young adulthood revealed a significant relationship (*F* = 5.05, *df* = 1, 124.2, *p* = 0.026) such that high-risk offspring with a lifetime history of internalizing disorder displayed larger corpus medullare volume (16.27 ± 0.51) relative to HR participants without a lifetime history of internalizing disorder (15.4 ± 0.24). However, within the LR group, the presence of a lifetime diagnosis of an internalizing disorder was associated with only minimal differences in the volume of the corpus medullare (14.87 ± 0.27 versus 14.01 ± 0.51).

**Table 4:** Externalizing psychopathology and volume of cerebellar lobes

	Corpus			Anterior			Superior posterior			Inferior posterior		
	*F*	*df*	*p*	*F*	*df*	*p*	*F*	*df*	*p*	*F*	*df*	*p*
Total volume												
Risk	1.20	1, 85.23	–	0.43	1, 80.38	–	0.76	1, 76.65	–	1.48	1, 72.18	–
Gender	26.53	1, 117.86	0.000	14.24	1, 125.00	0.000	14.38	1, 111.13	0.000	36.73	1, 124.17	0.000
Age	1.79	1, 112.50	–	0.28	1, 100.99	–	0.64	1, 111.92	–	0.95	1, 97.19	–
Externalizing	0.00	1, 124.95	–	1.63	1, 118.70	–	0.02	1, 124.85	–	0.43	1, 119.87	–
Risk × externalizing	4.82	1, 124.51	0.030	0.81	1, 116.46	–	0.71	1, 124.97	–	1.62	1, 117.28	–
Total gray												
Risk	2.54	1, 80.31	–	0.21	1, 79.17	–	1.31	1, 75.82	–	0.49	1, 82.12	–
Gender	16.66	1, 122.99	0.000	12.38	1, 122.93	0.001	13.92	1, 105.70	0.000	45.90	1, 121.19	0.000
Age	8.50	1, 104.08	0.004	1.48	1, 104.07	–	0.75	1, 114.84	–	1.34	1, 108.26	–
Externalizing	0.06	1, 115.81	–	1.67	1,116.81	–	0.01	1, 122.39	–	0.06	1, 120.64	–
Risk × externalizing	0.51	1, 113.79	–	0.63	1, 114.72	–	0.33	1, 122.95	–	2.58	1, 119.09	–
Total white												
Risk	0.59	1, 81.06	–	0.40	1, 79.87	–	1.60	1, 76.83	–	3.39	1, 123.00	–
Gender	20.71	1, 120.03	0.000	4.27	1, 119.60	0.041	2.81	1, 121.90	–	0.51	1, 123.00	–
Age	0.37	1, 108.75	–	3.40	1, 108.46	–	11.44	1, 100.73	0.001	2.89	1, 123.00	–
Externalizing	0.05	1, 121.36	–	1.57	1, 121.47	–	0.88	1, 110.98	–	0.31	1, 123.00	–
Risk × externalizing	4.92	1, 119.95	0.028	2.98	1, 120.05	–	0.34	1, 109.06	–	0.01	1, 123.00	–

**Table 5:** Internalizing psychopathology and volume of cerebellar lobes

	Corpus			Anterior			Superior posterior			Inferior posterior		
	*F*	*df*	*p*	*F*	*df*	*p*	*F*	*df*	*p*	*F*	*df*	*p*
Total volume												
Risk	11.55	1, 89.98	0.001	1.10	1, 87.87	–	4.54	1, 84.07	0.036	7.01	1, 74.77	0.010
Gender	27.08	1, 120.01	0.000	12.22	1, 125.00	0.001	15.85	1, 111.05	0.000	36.28	1, 124.60	0.000
Age	1.50	1, 107.55	–	0.14	1, 99.63	–	0.94	1, 109.00	–	0.87	1, 90.56	–
Internalizing	0.00	1, 124.97	–	0.91	1, 121.01	–	0.21	1, 122.36	–	0.02	1, 121.09	–
Risk × internalizing	5.05	1, 124.21	0.026	0.00	1, 116.24	–	2.63	1, 124.83	–	2.16	1, 114.80	–
Total gray												
Risk	6.04	1, 87.34	0.016	0.42	1, 86.32	–	5.33	1, 83.82	0.023	5.11	1, 87.14	0.026
Gender	17.91	1, 122.83	0.000	10.39	1, 122.97	0.002	15.99	1, 103.98	0.000	44.82	1, 121.71	0.000
Age	9.05	1, 102.69	0.003	1.17	1, 103.35	–	1.08	1, 112.76	–	1.44	1, 106.02	–
Internalizing	1.23	1, 115.38	–	1.12	1, 117.14	–	0.27	1, 120.35	–	0.22	1, 120.38	–
Risk × internalizing	1.19	1, 109.25	–	0.04	1, 111.23	–	2.83	1, 122.96	–	1.99	1, 115.65	–
Total white												
Risk	10.78	1, 84.29	0.001	0.04	1, 83.19	–	0.65	1, 87.87	–	4.91	1, 123	0.029
Gender	21.90	1, 121.73	0.000	2.57	1, 121.18	–	2.90	1, 122.10	–	0.63	1, 123	–
Age	0.21	1, 104.19	–	2.80	1, 103.97	–	12.44	1, 101.72	0.001	2.99	1, 123	–
Internalizing	0.26	1, 119.88	–	1.15	1, 120.33	–	0.09	1, 113.70	–	0.35	1, 123	–
Risk × internalizing	5.73	1, 114.56	0.018	0.01	1, 115.07	–	0.00	1, 107.33	–	0.69	1, 123	–

## Discussion

The present analyses revealed that high-risk offspring display regional differences in volume that are specific to particular cerebellar lobes. Familial risk for alcohol dependence was associated with larger volume of the inferior posterior lobe as well as the corpus medullare. In contrast, volume of the cerebellar lobes was not significantly associated with the presence of a previous substance use disorder. These results support the hypothesis that aberrant cerebellar morphology may represent a heritable premorbid marker of addiction risk. Individuals with a family history are at significantly higher risk for the development of substance use disorders than the general population [41, 42, 44]. These individuals have been found to display a variety of cognitive, affective, and temperamental characteristics that increase their vulnerability to developing alcohol and substance use problems, including impulsivity, deficits in emotion regulation, and poor cognitive control [31]. It has been hypothesized that these traits reflect heritable alterations in neural structure and function that drive the heighted addiction liability observed among high-risk offspring [31]. A better understanding of the neurobiological basis of risk for substance use disorders can provide insight into the etiology of these conditions and guide future intervention efforts.

High-risk offspring display neuropsychological deficits in a number of domains, including attention, language, response inhibition, and problem solving [30, 31, 45]. The current study also found poorer working memory performance among individuals with a dense family history of alcoholism relative to low-risk participants, in agreement with earlier reports [32, 33, 46]. Neuropsychological deficits have been found to prospectively predict alcohol and substance use and alcohol-related problems [47]. Additionally, abnormalities in the structure [22, 26] and function [48, 49] of prefrontal cortical regions known to support these functions have also been observed among high-risk offspring, and prefrontal activation during cognitive task performance has been identified as a prospective predictor of substance use [50–52].

Although cognitive functioning has been traditionally thought to rely predominantly on the prefrontal cortex, there is substantial evidence that the cerebellum also plays a critical role in cognition [53, 54]. Reciprocal projections between the cerebellum and prefrontal cortex have been identified that are thought to facilitate interactive effects of these regions on cognition [2, 53, 55]. Supporting these findings are observations that co-activation of the prefrontal cortex and cerebellum are seen during a wide variety of cognitive tasks [54]. Additionally, there is data to suggest a functional topography of the cerebellum, with posterior regions preferentially involved in cognitive processes whereas anterior portions of the cerebellum support motor functions [56]. Previous work comparing 17 alcohol-dependent individuals and 31 controls has revealed performance deficits in visuospatial working memory tasks that are related to structural differences in left cerebellar Crus I, though differences in verbal working memory functioning did not appear to be impaired in alcohol-dependent individuals [9]. In a study of 15 patients with spinocerebellar ataxia, verbal working memory deficits were found using the WAIS subtests (Forward and Backward Digit Span and Letter Number Sequencing) [10]. These deficits were significantly associated with gray matter density in superior cerebellar regions (bilateral lobules VI and Crus I of lobule VII) and inferior regions (bilateral lobules VII and right lobule IX). Using a sample of 311 healthy controls, performance on visuospatial working memory tasks has been shown to correlate with tissue volume in the left lobule VI and Crus I also suggesting that greater tissue volume is associated with better behavioral performance [11]. Interestingly, positive correlations were observed for the reaction time measure associated with performance of this visuospatial working memory task suggesting that greater gray matter volume may also be involved in less efficient processing [11]. fMRI studies have also demonstrated a relationship between the superior and inferior cerebellar lobes in normal controls using both verbal and visuospatial working memory paradigms that appear to show good reliability with respect to structural functional relationships [12–14]. In accordance with earlier findings, the current report also found positive correlations between volume of the superior and inferior posterior cerebellar lobes and working memory performance, controlling for both risk and gender.

A comment is needed regarding our findings showing a positive correlation between working memory performance and volumes of the corpus medullare, superior posterior, and inferior posterior lobes for the entire sample of high- and low-risk offspring. In view of the poorer working memory performance seen in high-risk offspring along with larger volumes of corpus medullare and the inferior posterior lobes, it may seem unexpected that positive partial correlations were found for the corpus medullare, superior posterior lobes, and inferior posterior lobes. Analyses within risk groups indicate that normal controls show the expected positive correlations but high-risk individuals do not. Overall, the high-risk offspring had larger volumes and poorer working memory performance indicating an expected negative correlation. However, due to variation within the high-risk sample, a significant relationship was not seen.

Based on the importance of the cerebellum in cognitive functions known to be impaired among children of alcoholics, there was reason to investigate whether premorbid cerebellar abnormalities are present among high-risk offspring that could influence addiction risk. Previous analyses from this laboratory show increased total volume of the cerebellum [20, 21]. These results suggested the importance of obtaining volumetric measures of individual cerebellar lobes to determine if this tendency for larger volume was uniform across the lobes. The present analysis showed significant risk group differences for the inferior posterior lobe as well as the corpus medullare, with high-risk offspring displaying larger volumes. This risk effect remained significant when controlling for gender, age, prior substance use disorder, as well as internalizing and externalizing psychopathology.

These data are consistent with the hypothesis that high-risk offspring display delayed trajectories of brain maturation [21, 31, 57]. In typical development, volume of the inferior posterior cerebellar lobe peaks at age 11 in females and at 13.5 years in males which is then followed by a protracted decline [29]. This pattern is consistent with normal age-related reductions in gray matter that take place throughout adolescence and early adulthood [58]. This reduction in gray matter volume has been linked to better cognitive functioning across a variety of domains [59]. In the current study, we observed larger volume of the corpus medullare and inferior posterior cerebellar lobe among individuals with a family history of alcohol dependence. Other studies contrasting cannabis users to non-users report finding larger regional gray matter volume of the anterior cerebellum [60] though family history of substance use disorders was not assessed. Similarly, adolescent cannabis users show significantly larger inferior posterior (lobules VIII–X) vermis volume than controls while alcohol use was associated with smaller posterior inferior volume [61]. Although family history of substance use disorders was not assessed in the Houston et al. study [59], it is possible that the larger volume seen among these cannabis users may reflect family history differences in susceptibility for substance use disorders.

One explanation for the larger volume of the inferior posterior lobes in high-risk subjects seen in the present study could be that high-risk offspring appear to reach their peak volume in these regions later [21]. Future longitudinal research is needed to confirm whether the larger volumes observed here reflect altered developmental trajectories. Delayed maturation of cerebellar lobes that support cognitive functions has important implications for understanding the neuropsychological deficits typical of high-risk children and may contribute to their heightened addiction vulnerability. The smaller volume of the corpus medullare which includes the central white matter and the output nuclei of the cerebellum seen in high-risk offspring with externalizing disorders is of interest.

Although there was no main effect of having a lifetime diagnosis that can be characterized as either an internalizing or externalizing disorder, significant interactions with familial risk were seen. Our findings concerning the association between volume of the corpus medullare and the presence of an internalizing or externalizing disorder are intriguing in view of previous reports of a cerebellar cognitive affective syndrome (CCAS) described by Schmahmann and Sherman [62]. This syndrome involves disturbances in executive functions, language difficulties, spatial cognition deficits, and personality changes. Cerebellar malformations have also been reported to increase the prevalence of a number of functional disabilities including cognitive, language, motor, and social behavioral deficits including internalizing and externalizing disorders in children [63] with rates of internalizing and externalizing disorders reaching 25 and 15 %, respectively, of the children identified [64].

### Limitations

One limitation of the current study is that some offspring had developed a substance use disorder, internalizing, or externalizing psychopathology prior to MRI scanning. In order to offset this problem, prior substance use was included as a covariate in the analyses of familial risk in order to differentiate risk and exposure effects. Notably, the influence of familial risk remained significant, and prior substance use disorder did not appear to be a significant predictor of cerebellar volume for any specific lobe. Analysis of the specific effects of anxiety, depression, conduct disorder, and ADHD could not be assessed due to insufficient number of participants with these individual conditions. However, when individual diagnoses were grouped into internalizing or externalizing disorders, the association between volumes of specific lobes and these diagnostic entities could be assessed. Although no main effects were observed for either class of psychopathology, an interaction with familial risk status was found for the corpus medullare. The findings suggest that the association between corpus medullare volume and presence of either class of disorders (internalizing or externalizing) is conditional on whether or not the participant has a multiplex family history of alcohol dependence. A further limitation was that approximately half of the sample had a lifetime history of having both an internalizing and externalizing disorder so analyses relating externalizing and internalizing psychopathologies to cerebellar lobe volumes could be assessed in only a reduced number of cases where only one constellation of disorders was present.

Another potential limitation is that some offspring were exposed to alcohol, drugs, or cigarettes in utero. However, the rate of alcohol and substance use during pregnancy in this sample is quite low and similar to the general population [21]. Therefore, the effect of familial risk on morphology of the cerebellar lobes is likely not attributable to prenatal exposures.

Additionally, the present report is based on a single MRI evaluation so that the developmental growth trajectories of our regions of interest could not be directly assessed. Future research is needed to quantify cerebellar lobe volumes at multiple time points to conclusively determine whether high- and low-risk offspring differ in their rate or pattern of cerebellar lobe maturation. Therefore, longitudinal investigations are necessary to fully characterize risk effects on cerebellar lobe structure and development.

## Conclusions

This is the first study to demonstrate regionally specific differences in cerebellar morphology among high-risk offspring. Our findings indicate that individuals with a family history of alcohol dependence exhibit larger volumes of the inferior posterior lobe and the corpus medullare, which may reflect a developmental delay in cerebellar brain development. These differences may reflect a slower rate of gray matter pruning in the inferior posterior lobe that, in turn, has implications for working memory functioning. Reduced volume of the corpus medullare in high-risk subjects with a history of externalizing disorder suggests that there may be reduced functional output to regions of the cerebrum (e.g., prefrontal cortex) that may influence executive functioning. Potential associations between frontocerebellar circuitry, volumes of specific cerebellar lobes, and executive functioning have previously been investigated in alcohol-dependent individuals in comparison with age-matched controls, with results showing that working memory strategies may differ between the groups that also reflect structural differences. The present findings further elucidate how altered cerebellar morphology may influence addiction risk. Although this study did not investigate specific genetic polymorphisms with respect to cerebellar lobe morphology, a previous study from this laboratory has found that variation in GABRA2 and BDNF alter total cerebellar volume [65].
